# Elevated X-Box Binding Protein1 Splicing and Interleukin-17A Expression Are Associated With Active Generalized Vitiligo in Gujarat Population

**DOI:** 10.3389/fimmu.2021.801724

**Published:** 2022-01-03

**Authors:** Shahnawaz D. Jadeja, Jayvadan Vaishnav, Ankit H. Bharti, Rasheedunnisa Begum

**Affiliations:** ^1^ Department of Biochemistry, Faculty of Science, The Maharaja Sayajirao University of Baroda, Vadodara, India; ^2^ Dermatology Department, Dr. Ankit’s Dermatopathology Research Centre, Vyara, India

**Keywords:** vitiligo, interleukin, endoplasmic reticulum stress (ER stress), genetic polymorphisms and disease association, autoimmunity, cytokines

## Abstract

Vitiligo is an autoimmune skin disorder defined by the destruction of functional epidermal melanocytes. It is a multifactorial and polygenic disorder caused due to oxidative stress, endoplasmic reticulum (ER) stress, and autoimmunity, among other factors. In the present study, we aimed to investigate the association of X-box Binding Protein 1 (XBP1) and Interleukin-17A (IL-17A) polymorphisms and monitor their systemic as well as skin expression levels in vitiligo patients from Gujarat population in India. *XBP1* rs2269577 G/C, *IL17A* rs2275913 G/A and *IL17A* rs8193036 C/T polymorphisms were genotyped by Polymerase Chain Reaction-Restriction Fragment Length Polymorphism (PCR-RFLP) method in 312 controls and 276 vitiligo patients. Transcript levels of spliced (*sXBP1*), unspliced *XBP1* (*uXBP1*) and *IL17A* from peripheral blood mononuclear cells (PBMCs) as well as spliced and unspliced *XBP1* from skin samples were analyzed by qPCR. IL-17A protein levels in suction-induced blister fluid (SBF) from the skin of study subjects were estimated by ELISA. The results revealed that genotype (*p*=0.010) and allele (*p*=0.014) frequencies of *XBP1* rs2269577 G/C polymorphism were significantly different, however, no significant difference was observed in frequencies of *IL17A* rs2275913 G/A and *IL17A* rs8193036 C/T polymorphisms in control and patient population. Gene expression analysis revealed that *sXBP1* and *IL17A* levels were significantly higher in PBMCs of generalized (*p*=0.030 and *p*=0.039, respectively) and active (*p*=0.024 and *p*=0.017, respectively) vitiligo patients. Moreover, we observed a significantly elevated *sXBP1* expression (*p*=0.037) as well as IL-17A protein levels (*p*=0.009) in perilesional skin of vitiligo patients as compared to controls. Overall, these findings suggest XBP1 and IL17A play an important role in vitiligo and further substantiate the involvement of ER stress in exacerbating immune-mediated vitiligo pathogenesis.

## Introduction

Vitiligo is a hypo-pigmentary disorder caused because of the destruction of epidermal melanocytes. The exact etiopathogenesis of vitiligo remains unknown despite extensive research. Our previous study in Gujarat revealed that around 22% of vitiligo patients demonstrated a familial association and about 14% of the patients had at least one first-degree relative with vitiligo (1). It has been reported that out of the total risk of vitiligo, about 20% is conferred by environmental factors, 57% by common genetic variants, and 23% by rare genetic variants (2). Currently, more than 50 vitiligo susceptibility loci have been identified in Caucasian population (3). Various susceptibility loci in the genes attributed to immunoregulation, cytokines and redox homeostasis have been conferred as predisposition loci for vitiligo in Gujarat population (4–18). Genetic polymorphisms might influence gene expression or protein function and thereby predispose individuals to dysregulation of the normal homeostasis leading to the onset of the disease. Based on several indications, earlier we proposed that endoplasmic reticulum (ER) stress could be one of the major events responsible for the trigger and progression of the disease through regulating various immunomodulatory mechanisms (19, 20). ER stress activates the unfolded protein response (UPR) *via* activating three transmembrane sensors viz. Inositol Requiring Enzyme-1 (IRE-1), Activating transcription factor 6 (ATF-6), and protein kinase RNA-like endoplasmic reticulum kinase (PERK) (21). X-box Binding Protein1 (XBP1) is involved in the downstream of IRE1 activation in the ER stress induced UPR mechanism (22). IRE1 oligomerizes and activates its ribonuclease domain through auto-phosphorylation. Activated IRE1 leads to the non-canonical splicing of a 26-nucleotide sequence from ubiquitously expressed *XBP1* mRNA (unspliced). Elimination of this intron triggers a frameshift in the *XBP1* mRNA resulting in the translation spliced XBP1 (sXBP1) isoform (23, 24). The sXBP1 is an active transcription factor that upregulates target genes *via* the ER stress-responsive element (ERSE) region. Apart from its known role in UPR, *XBP1* is also implicated in controlling plasma cell differentiation and immunity (25). *XBP1* -116 G/C polymorphism is located in the promoter of the *XBP1* gene affecting its promoter activity (26). *XBP1 -116 G/C* promoter polymorphism was associated with diabetes, inflammatory bowel disease, and bipolar disorder (27–29). *XBP1* is also reported to be associated with regulating various pro-inflammatory cytokines including IL-17A (30, 31). IL-17A is a pro-inflammatory cytokine produced by Th17 cells, which form a separate subset of the CD4^+^ T-cell lineage (32). Over recent years IL-17A has garnered the interest of several researchers due to its association with several autoimmune skin inflammatory disorders (33, 34). A previous study has reported elevated IL-17A levels in vitiliginous skin and serum of patients (35). Another study showed a positive link between serum IL-17A levels and the area of vitiligo lesions, indicating that Th17 cells are involved in the progression of vitiligo (36). Interestingly, it was found that IL-17A can stimulate the keratinocytes and fibroblasts to secrete TNF-α (37). Two polymorphisms in the *IL17A* promoter region (-197 G/A and -737 C/T) are reported to be associated with various disorders (38–41). Hence, in this study, we aimed to investigate the involvement of XBP1 and IL-17A in vitiligo susceptibility in Gujarat population.

## Methods

### Recruitment of Study Subjects

The study was approved by the Institutional ethical committee for human research (IECHR), Faculty of Science, The Maharaja Sayajirao University of Baroda, Vadodara, Gujarat, India (FS/IECHR/BC/RB/1). Total 312 matched controls and 276 vitiligo patients were enrolled in the present study from Gujarat population (**Table S1**). The inclusion and exclusion criteria, diagnosis of vitiligo and classification were performed, as described earlier (18). The study plan and aims were explained and written consent was obtained from all the participants.

### Blood and Skin Sample Collection

5ml blood was collected in K_3_EDTA tubes. The plasma fraction was used for ELISA assays and the cell fraction was used for DNA and RNA extraction. Skin biopsy samples were collected from selected controls and vitiligo patients without systemic immunosuppressive treatment or PUVA/UVB for at least 1 month, and topical therapy for at least 2 weeks. 4-mm punch biopsies were taken and snap-frozen from the lesional and non-lesional skin of patients with vitiligo, and non-inflamed, non-irritated skin of healthy individuals.

### DNA Isolation

DNA was isolated from peripheral blood mononuclear cells (PBMCs) using the phenol-chloroform extraction method and stored at -20°C after determining the concentration and quality. SNP genotyping was carried out by polymerase chain reaction-restriction fragment length polymorphism (PCR-RFLP) method as described in the ‘**Supporting Information**’ file.

### Gene Expression Analysis From the Blood and Skin Samples

Total RNA from PBMCs and skin biopsies were isolated using Trizol^®^ reagent (Invitrogen, Carlsbad, CA, USA). cDNA synthesis was carried out using the High-Capacity cDNA Reverse Transcription Kit (Applied Biosystems™, USA), as per the manufacturer’s instructions. Transcript level analyses of spliced & unspliced *XBP1, IL17A*, and *GAPDH* were carried out using qPCR. (Details provided in the ‘**Supporting Information**’ file).

### Collection of Suction-Induced Blister Fluid Samples

Suction-induced blister fluid samples were collected from the skin of controls (n=18) and active generalized vitiligo patients (n=15) with the help of an expert dermatologist (**Figure S1**). The povidone-iodine solution was applied to the suction area, which was then locally anesthetized by the infiltration of 2% lignocaine. Suction blisters were induced by applying the bases of the sterile disposable 20 ml syringes whose piston was removed, and their nozzles were connected to other 50 ml syringes to generate suction, then the infusion set valve was closed to maintain a negative suction. After a unilocular blister was obtained, the fluid was collected by a 2ml syringe and centrifuged at 3000rpm for 5 min at 4°C. The blister fluid samples were then stored at -80°C until the time of analysis.

### Estimation of IL-17A Cytokine Levels by ELISA

IL-17A protein levels were measured from suction induced blister fluid samples of the skin by using the human IL-17A ELISA Kit (KB1079; KRISHGEN Biosystems, India) as per the manufacturer’s instructions.

### Statistical Analyses

Analysis of Hardy-Weinberg equilibrium (HWE) for all the SNPs was carried out by comparison of the observed and expected frequencies of the genotypes using chi-square analysis. Genotype and allele frequency distribution in different groups were compared using the chi-square test with 2×2 contingency tables considering the major genotype/allele as reference. For genetic analysis, Bonferroni’s correction for multiple testing was applied and level of significance was considered at *p* ≤ 0.017. Odds ratio (OR) with 95% confidence interval (CI) for disease susceptibility was also calculated. The statistical power of the study was determined by G Power software (42) considering 0.2 effect size which achieved 99.8% power to detect the association of *XBP1* rs2269577 G/C polymorphism in the present study. For *IL17A* rs2275913 G/A polymorphism effect size 0.11 achieved 74.0% power and for *IL17A* rs8193036 C/T polymorphism effect size 0.13 achieved 87.5% power to detect the association of respective polymorphisms in the present study. For analyses of the transcript and protein levels, unpaired t-test and one-way ANOVA were performed as applicable. The level of significance for these analyses was considered at *p* < 0.05. Tukey’s multiple correction was applied for multiple testing and the *p* values were adjusted. All the statistical tests were carried out using Prism 6 software (Graph Pad Software, USA).

## Results

### *XBP1*-116 G/C (rs2269577) Polymorphisms With Vitiligo Susceptibility

Three genotypes were identified for *XBP1* rs2269577 polymorphism (**Figure S2**). Genotyping and allele distribution for *XBP1* rs2269577 polymorphism revealed that the study populations (controls and patients) were in accordance with the HWE (*p*=0.979 and *p*=0.288, respectively). Genotype ‘GG’ and allele ‘G’ were considered as references. The frequency of the ‘CC’ genotype was significantly higher in patients in comparison to controls (33% vs. 27% respectively, *p*=0.010) and was detected as the risk genotype (OR=1.88; **Table 1**). The variant allele ‘C’ was also found to be significantly higher in patients as compared to controls (59% vs. 52% respectively, *p*=0.014) and was detected as a risk allele (OR=1.34; **Table 1**). Further, based on the type of vitiligo, a predominant increase in the frequency of ‘CC’ genotype was observed in patients with Generalized vitiligo (GV) (*p*<0.0001) and Localized vitiligo (LV) (*p*<0.0001) in comparison to controls (**Table 2**). Further, with respect to the activity of the disease, revealed a significant difference in the frequency of ‘CC’ genotype in patients with Active vitiligo (AV) (*p*<0.0001) and Stable vitiligo (SV) (*p*<0.0001) as compared to controls (**Table 2**).

**Table 1:** Distribution of genotype and allele frequencies of *XBP1* rs2269577, *IL17A* rs2275913 and *IL17A* rs8193036 polymorphisms in vitiligo patients and controls.

SNP	Genotype or Allele	Controls (Freq) n=312	Patients (Freq) n=276	*p* for HWE	*p* value for association	OR	95% CI
XBP1 -116 G/C (rs2269577)	GG	72 (0.23)	42 (0.15)	0. 979 (C) 0.288 (P)	R	1	–
	GC	156 (0.50)	142 (0.52)		0.048^a^	1.56	1.00-2.43
	CC	33 (0.27)	92 (0.33)		0.010^a^	1.88	1.16-3.04
	G	300 (0.48)	226 (0.41)		R	1	–
	C	324 (0.52)	326 (0.59)		0.014^b^	1.34	1.06-1.68
IL17A -197 G/A (rs2275913)	GG	220 (0.70)	190 (0.69)	0.063 (C) 0.002 (P)	R	R	–
	GA	89 (0.29)	86 (0.31)		0.534^a^	1.12	0.78-1.59
	AA	3 (0.01)	0 (0.00)		0.109^a^	0.16	0.008-3.22
	G	529 (0.85)	466 (0.84)		R	R	–
	A	95 (0.15)	86 (0.16)		0.866^b^	1.03	0.75-1.41
IL17A -737 C/T (rs8193036)	CC	90 (0.29)	80 (0.29)	0.794 (C) 0.041 (P)	R	R	–
	CT	153 (0.49)	121 (0.44)		0.551^a^	1.89	0.60–1.31
	TT	69 (0.22)	75 (0.27)		0.375^a^	1.22	0.78–1.90
	C	333 (0.53)	281 (0.51)		R	R	–
	T	291 (0.47)	271 (0.49)		0.399^a^	1.10	0.88–1.39

‘n’ represents number of Patients/Controls, ‘R’ represents the reference group, HWE refers to Hardy-Weinberg Equilibrium, CI refers to Confidence Interval, Odds ratio is based on allele frequency distribution. (P) refers to Patients and (C) refers to Controls.

a Vitiligo Patients vs. Controls (genotype) using the chi-squared test with 2×2 contingency table.

b Vitiligo Patients vs. Controls (allele) using the chi-squared test with 2×2 contingency table.

**Table 2:** Distributions of genotype and allele frequencies of *XBP1* rs2269577, *IL17A* rs2275913 and *IL17A* rs8193036 polymorphisms in different subsets of vitiligo patients and controls.

SNP	Genotype or Allele	Controls n=312	GV n=204	LV n=72	*p* value	AV n=198	SV n=78	*p* value
XBP1 -116 G/C (rs2269577)	GG	72 (0.23)	29 (0.14)	13 (0.18)	R	32 (0.16)	10 (0.13)	R
	GC	156 (0.50)	113 (0.55)	29 (0.40)	0.153^a^ 0.019^b^ 0.936^c^	98 (0.50)	44 (0.56)	0.369^x^ 0.163^y^ 0.057^z^
	CC	33 (0.27)	62 (0.31)	30 (0.42)	0.849^a^ <0.0001^b^ <0.0001^c^	68 (0.34)	24 (0.20)	0.779^x^ <0.0001^y^ <0.0001^z^
	G	300 (0.48)	171 (0.42)	55 (0.38)	R	162 (0.41)	64 (0.41)	R
	C	324 (0.52)	237 (0.58)	89 (0.62)	0.435^a^ 0.052^b^ 0.032^c^	234 (0.59)	92 (0.59)	0.980^x^ 0.025^y^ 0.114^z^
IL17A -197 G/A (rs2275913)	GG	220 (0.70)	130 (0.64)	52 (0.72)	R	127 (0.64)	48 (0.62)	R
	GA	89 (0.29)	74 (0.36)	20 (0.28)	0.191^a^ 0.075^b^ 0.862^c^	71 (0.36)	30 (0.38)	0.686^x^ 0.095^y^ 0.099^z^
	AA	03 (0.01)	0 (0.0)	0 (0.0)	-^a^ 0.184^b^ 0.400^c^	0 (0.0)	0 (0.0)	-^a^ 0.189^b^ 0.419^c^
	G	529 (0.58)	334 (0.82)	124 (0.86)	R	325 (0.82)	126 (0.81)	R
	A	95 (0.15)	74 (0.18)	20 (0.14)	0.243^a^ 0.216^b^ 0.686^c^	71 (0.18)	30 (0.19)	0.722^x^ 0.254^y^ 0.222^z^
IL17A -737 C/T (rs8193036)	CC	90 (0.29)	59 (0.29)	24 (0.33)	R	57 (0.29)	26 (0.33)	R
	CT	153 (0.49)	90 (0.44)	31 (0.43)	0.602^a^ 0.612^b^ 0.363^c^	81 (0.41)	40 (0.51)	0.795^x^ 0.411^y^ 0.726^z^
	TT	69 (0.22)	55 (0.27)	17 (0.24)	0.455^a^ 0.427^b^ 0.824^c^	60 (0.30)	12 (0.15)	0.034^x^ 0.194^y^ 0.183^z^
	C	333 (0.53)	208 (0.51)	79 (0.55)	R	195 (0.49)	92 (0.59)	R
	T	291 (0.47)	200 (0.49)	65 (0.45)	0.423^a^ 0.453^b^ 0.746^c^	201 (0.51)	64 (0.41)	0.039^x^ 0.199^y^ 0.208^z^

n, number of subjects; R, reference group; GV, Generalized vitiligo; LV, Localized vitiligo; AV, Active Vitiligo; SV, Stable Vitiligo.

a Generalized vitiligo vs. Localized vitiligo.

b Generalized vitiligo vs. Controls.

c Localized vitiligo vs. Controls.

x Active Vitiligo vs. Stable Vitiligo.

y Active Vitiligo vs. Controls.

z Stable Vitiligo vs. Controls using the chi-squared test with 2 × 2 contingency table.

### Analysis of Unspliced and Spliced *XBP1* Transcript Levels in PBMCs

**Figure 1:**
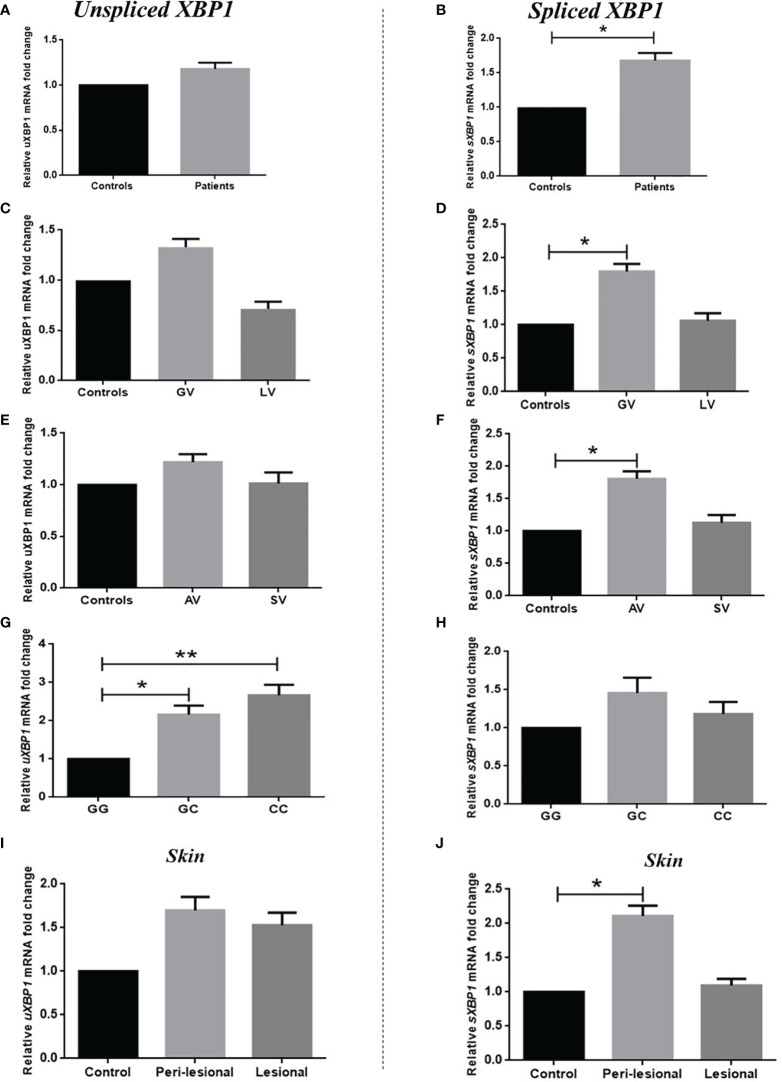
Analysis of *uXBP1* and *sXBP1* in PBMCs and skin samples of vitiligo patients and controls. Expression of *uXBP1* and *sXBP1* transcript levels in PBMCs of 106 controls, 103 patients with vitiligo were analysed by applying unpaired t-test for comparison between two groups and one-way ANOVA for comparison among three groups. **(A)** No significant difference in *uXBP1* transcript levels was observed among patients and controls (mean ΔCt ± SEM: 5.76 ± 0.210 vs 5.98 ± 0.204 respectively; *p*=0.456). Expression fold change of *uXBP1* transcripts in patients against controls showed a 1.18-fold higher expression as determined by the 2^-ΔΔCp^ method. **(B)** Transcript levels of *sXBP1* were significantly different among patients and controls (mean ΔCt ± SEM: 4.61 ± 0.187 vs. 5.28 ± 0.229, respectively; *p*=0.026). Expression fold change of *sXBP1* transcripts in patients against controls showed 1.76-fold higher expression as determined by the 2^-ΔΔCp^ method. Expression of *uXBP1* and *sXBP1* transcripts were further analysed in 106 controls and 87 patients with GV and 16 patients with LV. **(C)** No significant difference in *uXBP1* transcripts was observed in patients with GV and LV as compared to controls (*p*=0.404 and *p*=0.610, respectively). Further, *uXBP1* transcript levels were not significantly different among patients with GV and LV (*p*=0.245). **(D)** Patients with GV showed significantly increased *sXBP1* transcript levels as compared to controls (*p*=0.030). However, there was no significant difference in *sXBP1* transcript levels between patients with GV and LV as well as in patients with LV as compared to controls (*p*=0.343 and *p*=0.999, respectively). Expression of *uXBP1* and *sXBP1* transcripts were also analysed in 106 controls and 83 patients with AV and 20 patients with SV. **(E)** No significant difference in *uXBP1* transcripts was observed in patients with AV and SV as compared to controls (*p*=0.683 and *p*=0.996, respectively). Further, *uXBP1* transcript levels were not significantly different among patients with GV and LV (*p*=0.843). **(F)** Patients with AV showed significantly increased *sXBP1* transcript levels as compared to controls (*p*=0.024). However, there was no significant difference in *sXBP1* transcript levels between patients with AV and SV as well as in patients with SV as compared to controls (*p*=0.408 and *p*=0.975, respectively). Expression of *uXBP1* and *sXBP1* transcripts were further analysed with respect to the *XBP1* rs2269577 polymorphism in 106 controls and 103 patients. **(G)** Individuals with GC and CC genotypes showed significantly increased *uXBP1* transcripts as compared to GG genotype (*p*=0.033 and *p*=0.007, respectively). No significant difference in *uXBP1* transcripts levels was observed in individuals with GC and CC genotypes (*p*=0.741). **(H)** No significant difference in *sXBP1* transcripts levels was observed in individuals with GC and CC genotypes as compared to the GG genotype (*p*=0.730 and *p*=0.969, respectively). Further, no significant difference was observed in *sXBP1* transcripts levels in individuals with the GC and CC genotypes (*p*=0.792). [*p<0.05; **p<0.01]. Analysis of *uXBP1* and *sXBP1* transcript levels in skin samples of 12 vitiligo patients and 15 controls were carried out by using one-way ANOVA **(I)** No significant difference in expression of *uXBP1* transcripts was observed in peri-lesional and lesional skin as compared to control skin (*p*=0.183 and *p*=0.496, respectively) as well as in peri-lesional skin as compared to lesional skin (*p*=0.805). **(J)** A significant increase in *sXBP1* transcript levels was observed in peri-lesional skin as compared to control skin (*p*=0.037; 2.26-fold) however, there was no significant difference in *sXBP1* transcript levels in lesional skin as compared to control skin (*p*=0.973) and among peri-lesional skin and lesional skin (*p*=0.071) [*p<0.05].

We monitored the levels of unspliced *XBP1 (uXBP1)* and spliced *XBP1 (sXBP1)* transcript levels in 106 controls and 103 patients. Analyses suggested no significant difference in the *uXBP1* transcript levels (*p*=0.456; **Figure 1A**). However, there was a significant difference in *sXBP1* transcript levels (*p*=0.026). The fold change analysis showed an approximate 1.76-fold increase in *sXBP1* transcript levels in patients (**Figure 1B**). Further, the analysis based on disease type and activity suggested that *sXBP1* transcript levels were increased significantly in GV as well as AV patients in contrast to controls (*p*=0.030 and *p*=0.024 respectively; **Figures 1D, F**), suggesting the involvement of ER stress in the immune-mediated vitiligo pathogenesis. Though, no significant difference in *uXBP1* transcript levels was observed upon an analysis based on disease type, or activity (**Figures 1C, E**). Furthermore, the *uXBP1* and *sXBP1* transcripts levels were analysed with respect to the *XBP1* rs2269577 polymorphism. The results suggest that the individuals with GC and CC genotypes had significantly higher *uXBP1* transcripts levels as compared to the GG genotype (*p*=0.033 and *p*=0.007, respectively). Whereas *uXBP1* transcripts levels were not significantly different for individuals carrying GC and CC genotypes (*p*=0.741; **Figure 1G**). However, *sXBP1* transcripts levels were not significantly different in individuals with GC and CC genotypes in comparison to the GG genotype (*p*=0.730 and *p*=0.969, respectively) and persons carrying the GC and CC genotypes (*p*=0.792; **Figure 1H**).

### Analysis of Unspliced and Spliced *XBP1* Transcript Levels in Skin Samples

Analysis of *uXBP1* and *sXBP1* transcript levels was performed in skin biopsies of 12 patients and 15 controls. The results did not show any significant difference in expression of *uXBP1* transcript levels in the skin samples of patients and controls (*p*=0.037; **Figure 1I**). Interestingly, *sXBP1* transcript levels were found to be significantly higher in the perilesional skin of vitiligo patients as compared to control skin (*p*=0.037). The fold change analysis showed about a 2.26-fold increase in *sXBP1* transcript levels in peri-lesional skin as compared to control skin, whereas the expression of *sXBP1* lesional skin was not significantly different as compared to control (*p*=0.973) and peri-lesional skin of vitiligo patients (*p*=0.071; **Figure 1J**).

### Investigating the Role of *IL17A* -197 G/A (rs2275913) and -737 C/T (rs8193036) Polymorphisms With Vitiligo Susceptibility

Three genotypes were identified for *IL17A* rs2275913 polymorphism (**Figure S3**). Analysis of genotype distribution revealed that the control population was following HWE (*p*=0.063) however patient population deviated from HWE (*p*=0.002). ‘GG’ genotype and ‘G’ allele were considered as the reference for further analysis. The genotype and allele frequencies in patient and control populations were not significantly different (**Table 1**). Further, analysis of *IL17A* rs2275913 polymorphism based on the type and activity of vitiligo revealed no significant difference in genotype and allele frequencies (**Table 2**).

Three genotypes were identified for *IL17A* rs8193036 polymorphism (**Figure S4**). Analysis of genotype distribution revealed that the control population was following HWE (*p*=0.793) whereas, the patient population deviated from HWE (*p*=0.041). ‘CC’ genotype and ‘C’ allele were considered as the reference for further analysis. The genotype and allele frequencies were not significantly different in patient and control populations (**Table 1**). *IL17A* rs8193036 polymorphism, when analysed based on the type of vitiligo and activity of the disease, no significant difference was observed in genotype and allele frequencies (**Table 2**).

### Analysis of *IL17A* Transcript Levels in PBMCs

**Figure 2:**
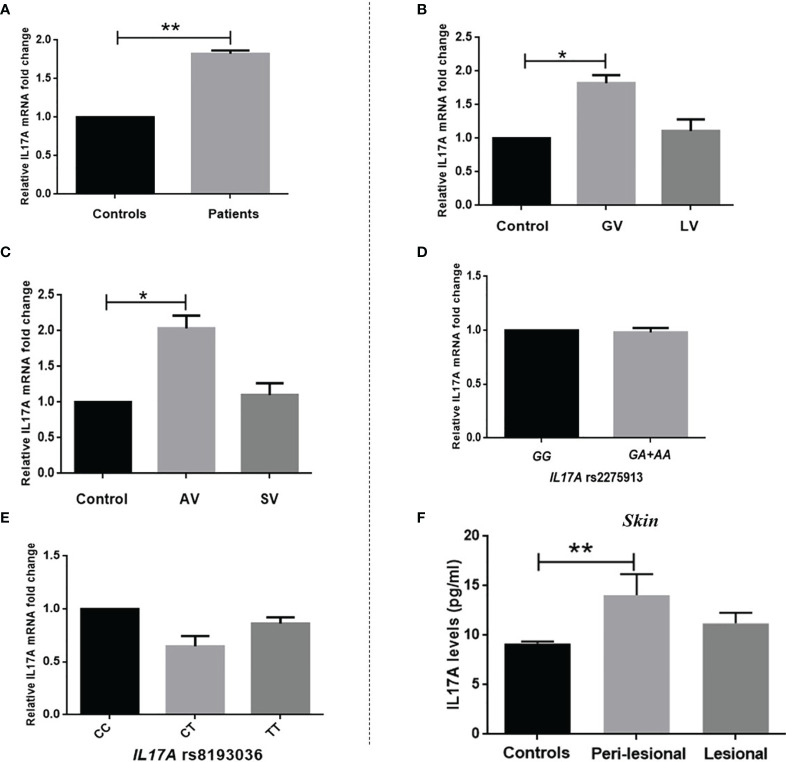
Relative gene expression of *IL17A* in PBMCs of vitiligo patients and controls. Expression of *IL17A* transcripts in PBMCs of 108 controls, 100 patients with vitiligo was analysed by applying unpaired t-test for comparison between two groups and one-way ANOVA for comparison among three groups. **(A)** Patients showed a significant increase in transcript levels of *IL17A* compared to controls (mean ΔCt ± SEM: 3.29 ± 0.244 vs. 4.16 ± 0.210; *p*=0.007, respectively). Expression of *IL17A* transcripts in patients against controls showed a 1.82-fold increase as determined by the 2^-ΔΔCp^ method. **(B)** Expression of *IL17A* transcripts in 108 controls and 84 patients with GV and 16 patients with LV was further analysed. Patients with GV showed significantly increased *IL17A* transcript levels as compared to controls (*p*=0.039). However, there was no significant difference in *IL17A* transcript levels between patients with GV and LV as well as in patients with LV as compared to controls (*p*=0.508 and *p*=0.975, respectively). **(C)** Expression of *IL17A* transcripts in 108 controls and 81 patients with AV and 19 patients with SV was analysed. Patients with AV showed significantly increased *IL17A* transcript levels as compared to controls (*p*=0.017). However, there was no significant difference in *IL17A* transcript levels between patients with AV and SV as well as in patients with SV as compared to controls (*p*=0.377 and *p*=0.979, respectively. **(D)** No significant difference was observed in *IL17A* transcript levels in individuals with GG as compared to those with GA+AA genotypes of *IL17A* rs2275913 polymorphism (*p*=0.944). **(E)** Further, individuals with the CC genotype of *IL17A* rs8193036 polymorphism did not show any significant difference in IL17A expression as compared to those with CT and TT genotypes (*p*=0.549 and *p*=0.719, respectively). [*p<0.05; **p<0.01]. **(F)** Estimation of IL-17A protein levels in SBF samples of patients with active generalized vitiligo (n=15) and controls (n=18). Significantly elevated IL-17A levels were observed in SBF samples from perilesional skin (*p*=0.009) of patients as compared to controls. No significant difference in IL-17A levels was observed in the lesional skin of patients as compared to peri-lesional (*p*=0.139) and control skin (*p*=0.266) [**p<0.01].

*IL17A* transcript level analyses revealed a significant increase in expression of *IL17A* transcripts in patients in comparison to controls (*p*=0.007). The fold change analysis showed an approximate 1.82-fold increase in the expression of *IL17A* transcript levels in patients as compared to controls (**Figure 2A**). Interestingly, the analysis based on the type and activity of the disease revealed a significant increase in *IL17A* transcript levels in patients with GV and AV as compared to controls (*p*=0.039 and *p*=0.017, respectively; **Figures 2B, C**). This finding suggested the association of IL17A with autoimmune vitiligo. Though, t IL17A transcript levels in patients with GV vs. LV and AV vs. SV were not significantly different as compared to controls (*p*=0.975 and *p*=0.979, respectively; **Figures 2B, C**). Expression of *IL17A* transcripts levels was further analysed with respect to *IL17A* rs2275913 and rs8193036 polymorphisms to assess the effect of SNPs on gene expression. The analysis with respect to *IL17A* rs2275913 polymorphism revealed no significant difference in *IL17A* transcript levels in individuals with GG as compared to those with GA+AA genotypes (*p*=0.944; **Figure 2D**). Similarly, *IL17A* transcript levels were not significantly different in individuals with the CC genotype as compared to those with CT and TT genotypes of *IL17A* rs8193036 polymorphism (*p*=0.549 and *p*=0.719, respectively **Figure 2E**).

### Analysis of IL-17A Protein Levels in Suction-Induced Blister Fluid (SBF) Samples

A significant increase in *IL17A* gene expression was observed in PBMCs of patients with generalized and active vitiligo. Hence, we monitored IL-17A levels in the skin of patients with active generalized vitiligo by ELISA (**Figure 2F**). The results suggest a significant increase in IL-17A levels in SBF samples from peri-lesional skin of active generalized vitiligo patients as compared to controls (mean ± SEM: 14.06 ± 2.139 pg/ml vs. 9.17 ± 0.201 pg/ml, respectively; *p*=0.009). However, no significant difference was observed in IL-17A levels in SBF from lesional skin as compared to peri-lesional (mean ± SEM: 11.21 ± 1.058 pg/ml vs 14.06 ± 2.139 pg/ml, respectively; *p*=0.0430) and control (mean ± SEM: 11.21 ± 1.058 pg/ml vs 9.17 ± 0.201 pg/ml, respectively; *p*=0.0430).

## Discussion

The melanocyte destruction in vitiligo is a consequence of incomprehensible interactions of biochemical, environmental, and immunological events, in a predisposed genetic environment. Interestingly, ER stress has been implicated as a connection between oxidative stress and autoimmunity in vitiligo pathogenesis (19, 20). Perturbation in normal ER homeostasis due to internal or external stimuli that affect ER function leading to accumulation of misfolded proteins is known as ER stress. X-box binding protein-1 is a transcription factor, regulating the expression of genes necessary for the proper functioning of the immune system and in the cellular stress response mechanism (43). The analysis of *XBP1* rs2269577 polymorphism revealed a significant difference in allele as well as genotype frequencies among controls and patients (**Table 1**) suggesting its association with vitiligo. Interestingly, *XBP1* rs2269577 was revealed to be associated with vitiligo susceptibility in Chinese and Caucasian people (44, 45). Our results suggest a significant increase in ‘CC’ genotype in vitiligo patients as compared to controls, hence *XBP1* rs2269577 polymorphism leads to alteration in the motif ‘AGGT’ into ‘ACGT’ in the *XBP1* gene promoter (46). Analysis of luciferase activity revealed higher promoter activity of the ‘C’ allele as compared to the ‘G’ allele (44, 46). Our results on *XBP1* transcript levels also showed significantly increased transcript levels of *uXBP1* in individuals carrying ‘CC’ and ‘CG’ genotypes; however, s*XBP1* transcript levels were not significantly different concerning *XBP1* rs2269577 genotypes (**Figure 1**). Further, gene expression analysis revealed a substantial increase in *sXBP1*transcript levels in PBMCs of patients with generalized and active vitiligo (**Figure 1**), suggesting its involvement in immune-mediated pathogenesis. However, transcript levels of *uXBP1* were not significantly different in PBMCs of vitiligo patients and controls (**Figure 1**). In addition, there was a significant increase of *sXBP1* transcript levels in peri-lesional skin of vitiligo patients as compared to lesional and control skin (**Figure 1**). The peri-lesional skin of vitiligo patients is an immunologically active region having infiltration of cytotoxic T cells (47). Overall, the gene expression analysis in PBMCs and skin samples suggests the involvement of an active state of IRE1 in the immune-mediated pathogenesis of vitiligo. IRE1/XBP1 is the most preserved branch of the UPR. It plays a crucial role in several important biological pathways influencing development, metabolism, immunity, and inflammation (25). The active transcription factor XBP1 is involved in the regulation of several important immunological processes including MHC molecules, cytokine expression, development and differentiation of immune cells (25, 30, 48–50). These findings suggest a possible link between cellular stress and immunity in the pathogenesis of vitiligo. Brucklacher-Waldert et al. (31) have reported that XBP1 is involved in the differentiation of Th17 cells. This suggests that ER stress favouring microenvironment could lead to elevated expression of pro-inflammatory cytokines including IL-17A. Interestingly, Kotobuki et al. (37) have demonstrated that there is an infiltration of Th17 cells in vitiliginous skin consequently leading to melanocyte destruction in the epidermis. IL-17A, secreted by Th17 cells, has been widely associated with several immune-mediated disorders. In the present study, we did not observe a significant association of *IL17A* rs2275913 and rs8193036 polymorphisms with vitiligo predisposition in Gujarat (**Table 2**). Several studies have reported a significant association of *IL17A* rs2275913 and rs8193036 polymorphisms with inflammatory disorders such as inflammatory bowel disease, autoimmune thyroid disease, ulcerative colitis, rheumatoid arthritis, Behcet’s disease, etc. (40, 51–54). However, Mohammed et al. (55) have reported no significant association of *IL17A* rs2275913 with vitiligo susceptibility in the Egyptian population. The gene expression analysis revealed a significant increase in *IL17A* transcript levels in PBMCs of vitiligo patients as compared to controls (**Figure 2**). The analysis based on the type and activity of vitiligo revealed a significant increase in *IL17A* transcript levels in PBMCs of patients with generalized and active vitiligo (**Figure 2**). However, there was no significant difference in *IL17A* transcript levels with respect to *IL17A* rs2275913 and rs8193036 polymorphisms suggesting no significant effect of both the polymorphisms on *IL17A* expression. A significant increase in *IL17A* transcript levels in PBMCs of generalized and active vitiligo led us to monitor the expression of IL-17A in the skin of vitiligo patients and controls. Hence, we estimated the IL-17A protein levels in suction-induced blister fluid (SBF) samples of vitiligo patients and controls. Interestingly, there was a significant increase in IL-17A levels in peri-lesional skin SBF samples as compared to controls (**Figure 2**). The elevated IL-17A levels in the perilesional skin of vitiligo patients might be as a result of IL-17A secreting Th17 cells infiltrating the vitiliginous lesions. Our results substantiate the findings of Wang et al. (56) and Kotobuki et al. (37) who earlier reported the presence of infiltrating Th17 cells in vitiliginous skin. Particularly our results are supported by the findings of Wang et al. (56) who reported significantly increased expression of *IL17* in the leading edge (perilesional) of vitiligo skin biopsies. Several other studies have also reported a significant increase in IL-17A levels in the skin as well as blood samples of vitiligo patients (55, 57–60). In addition, a positive correlation between IL-17A levels and the extent of depigmentation has also been reported, suggesting its involvement in the development of the disease (36). Kotobuki et al. (37) demonstrated that IL-17A can induce the neighbouring keratinocytes and fibroblasts to secrete other pro-inflammatory cytokines such as TNFα, IL-6, and IL-1β. They further demonstrated the adverse effect of IL-17A on melanocyte homeostasis. A significant decrease in genes involved in melanogenesis such as *MITF, TYR, TYRP1*, and *DCT* was observed in primary human melanocytes treated with IL-17A affecting normal melanogenesis (37).

In conclusion, the present study identifies *XBP1* rs2269577 polymorphism as genetic susceptibility loci for vitiligo in Gujarat population. In addition, we report an increased *XBP1* splicing and *IL17A* expression in vitiligo patients. Overall, these findings suggest a potential role of ER stress in shaping autoimmunity in vitiligo.

## Data Availability Statement

The original contributions presented in the study are included in the article/**Supplementary Material**. Further inquiries can be directed to the corresponding author.

## Ethics Statement

The studies involving human participants were reviewed and approved by Institutional ethical committee for human research (IECHR), Faculty of Science, The Maharaja Sayajirao University of Baroda, Vadodara, Gujarat, India. The patients/participants provided their written informed consent to participate in this study.

## Author Contributions

RB and SJ conceived the idea. AB and SJ recruited the study subjects and collected the samples. SJ and JV performed the experiments. SJ acquired and analyzed the data. SJ has written the original draft. RB supervised the study, provided the necessary infrastructure, and contributed to the critical revision and approval of the manuscript. All authors contributed to the article and approved the submitted version.

## Conflict of Interest

The authors declare that the research was conducted in the absence of any commercial or financial relationships that could be construed as a potential conflict of interest.

## Publisher’s Note

All claims expressed in this article are solely those of the authors and do not necessarily represent those of their affiliated organizations, or those of the publisher, the editors and the reviewers. Any product that may be evaluated in this article, or claim that may be made by its manufacturer, is not guaranteed or endorsed by the publisher.
