# Supplementary material for: Elevated X-Box Binding Protein1 Splicing and Interleukin-17A Expression Are Associated With Active Generalized Vitiligo in Gujarat Population
Source: Front Immunol. 2022 Jan 3;12:801724. doi: 10.3389/fimmu.2021.801724 (PMC8761938; doi:10.3389/fimmu.2021.801724)
Supplement: Supplementary file 1 [file DataSheet_1.docx]

**Supplementary Information**

1. **Materials and Methods:**

***1.1 SNP genotyping by PCR-RFLP***

PCR-RFLP was used to genotype *XBP1* -116 G/C (rs2269577), *IL17A* -197 G/A (rs2275913) and -737 C/T (rs8193036) polymorphisms. The details of specific primers and restriction enzymes used are provided in Table S2. The reaction mixture of the total volume of 20 µL included 3 µL (100ng) of genomic DNA, 11 µL nuclease-free H_2_O, 2.0 µL 10x PCR buffer, 2 µL 2 mM dNTPs (PureGene^TM^, India), 1 µL of 10 pM corresponding forward and reverse primers (Eurofins^TM^, India), and 1 U of Taq Polymerase (PureGene^TM^, India). Amplification was performed in Eppendorf Mastercycler Gradient Thermocycler (Eppendorf^TM^, Germany) according to the protocol: 95°C for 10 minutes followed by 45 cycles of 95°C for 30 seconds, annealing for 30 seconds (Table S2) and 72°C for 30 seconds, and 72°C for 10 minutes. The amplified products were checked by electrophoresis on a 2.0% agarose gel stained with ethidium bromide. 15 µL of the amplified products were digested with 1U of respective restriction enzyme in a total reaction volume of 20 µL as per the manufacturer’s instruction. The digested products were resolved with a 50 bp DNA ladder (HiMedia^TM^, India) on 3.5% agarose gel stained with ethidium bromide and visualized under E-Gel Imager (Life Technologies^TM^, Carlsbad, CA). More than 10% of the samples were randomly selected for reconfirmation and the results were 100% concordant. PCR products for each genotype were amplified as mentioned above and outsourced for Sanger’s sequencing to Barcode Biosciences (Banglore, India). The results of Sanger’s sequencing were analysed with Chroma’s lite 2.1 software (Technelysium Pty Ltd.) and the results were concordant with PCR-RFLP method (Figure S5).

***1.2 Gene expression analysis by qPCR***

Transcript level analysis of selected candidate genes was carried out from PBMCs and skin samples using qPCR. Glyceraldehyde 3-phosphate dehydrogenase (*GAPDH)* gene was used as a reference gene. Primer details for the respective genes are provided in Table S2. Real-Time qPCR was performed in duplicates in 20 μl volume using Light Cycler^®^ 480 SYBR Green I Master Mix (Roche Diagnostics, GmbH, Mannheim, Germany) following the manufacturer’s instructions in the Light Cycler 480 Real-Time PCR (Roche Diagnostics GmbH, Mannheim, Germany). The thermal cycling conditions included an initial activation step at 95°C for 10 min, followed by 45 cycles of 95°C for 20 seconds, annealing for 20 seconds Table S2, and 72°C for 20 seconds. The fluorescence signals were captured during the extension step. At the end of the amplification phase, a melt curve analysis was carried out to check the specificity of the products formed. C_t_ values were determined by the first cycle number at which fluorescence was greater than the set threshold value. The ΔC_t_ value was determined as the difference between the cycle threshold of target genes and the reference gene (*GAPDH*). The difference between the two ΔC_t_ values (ΔC_t_ controls and ΔC_t_ patients) was considered as ΔΔC^t^ to obtain the value of fold expression (2^-ΔΔCt^).

- 1. ***Human IL-17A ELISA kit***

IL-17A protein levels were measured from suction induced blister fluid samples of the skin by using the human IL-17A ELISA Kit (KB1079; KRISHGEN Biosystems, India) with sensitivity of 15 pg/ml as per the manufacturer’s instructions.

1. **Supplementary Figures**


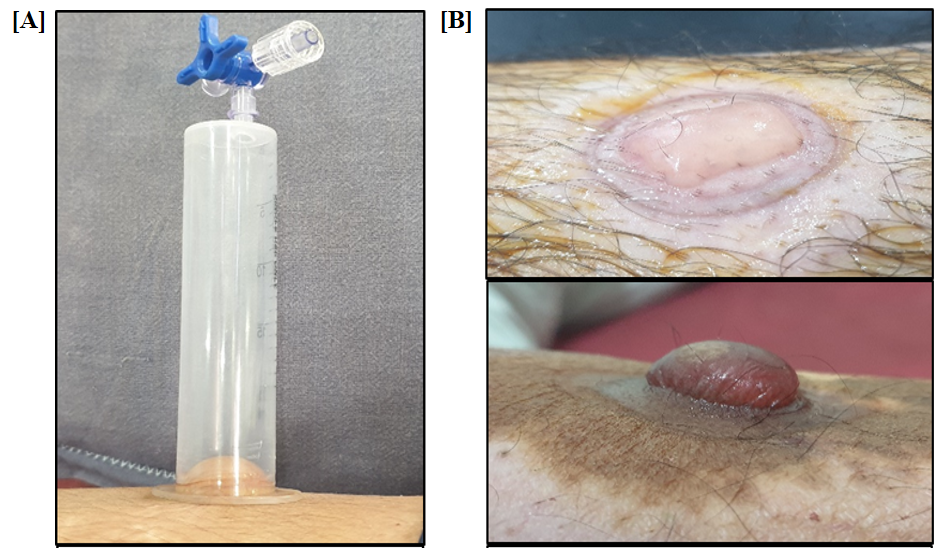


**Figure S1:** Representative images of [A] suction device and [B] suction induced blisters.


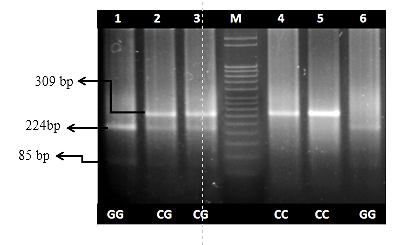


**Figure S2: PCR-RFLP analysis of *XBP1* rs2269577 polymorphism on 3.5 % agarose gel electrophoresis.** Lanes 2 & 3 show heterozygous (CG) genotype; lanes 1 & 6 show homozygous (GG) genotype; lanes 4 & 5 show homozygous (CC) genotype; lane M shows 50 bp DNA ladder.

**
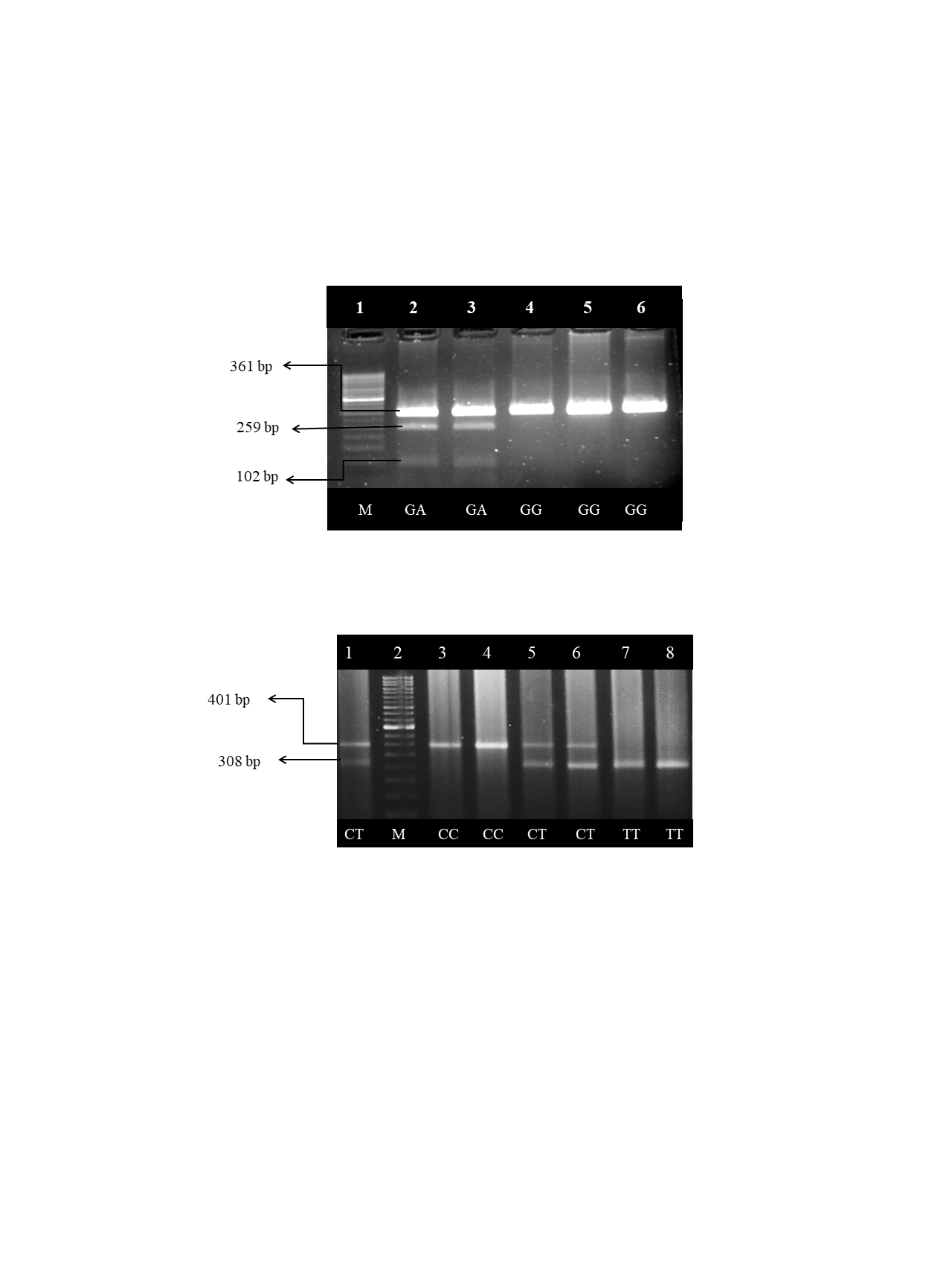
**

**Figure S3: PCR-RFLP analysis of *IL17A* rs2275913 polymorphism on 3.5 % agarose gel electrophoresis.** Lanes: 2 & 3 show heterozygous (GA) genotype; lanes: 4, 5 & 6 show homozygous (GG) genotype; lane 1 shows 50 bp DNA ladder.

**
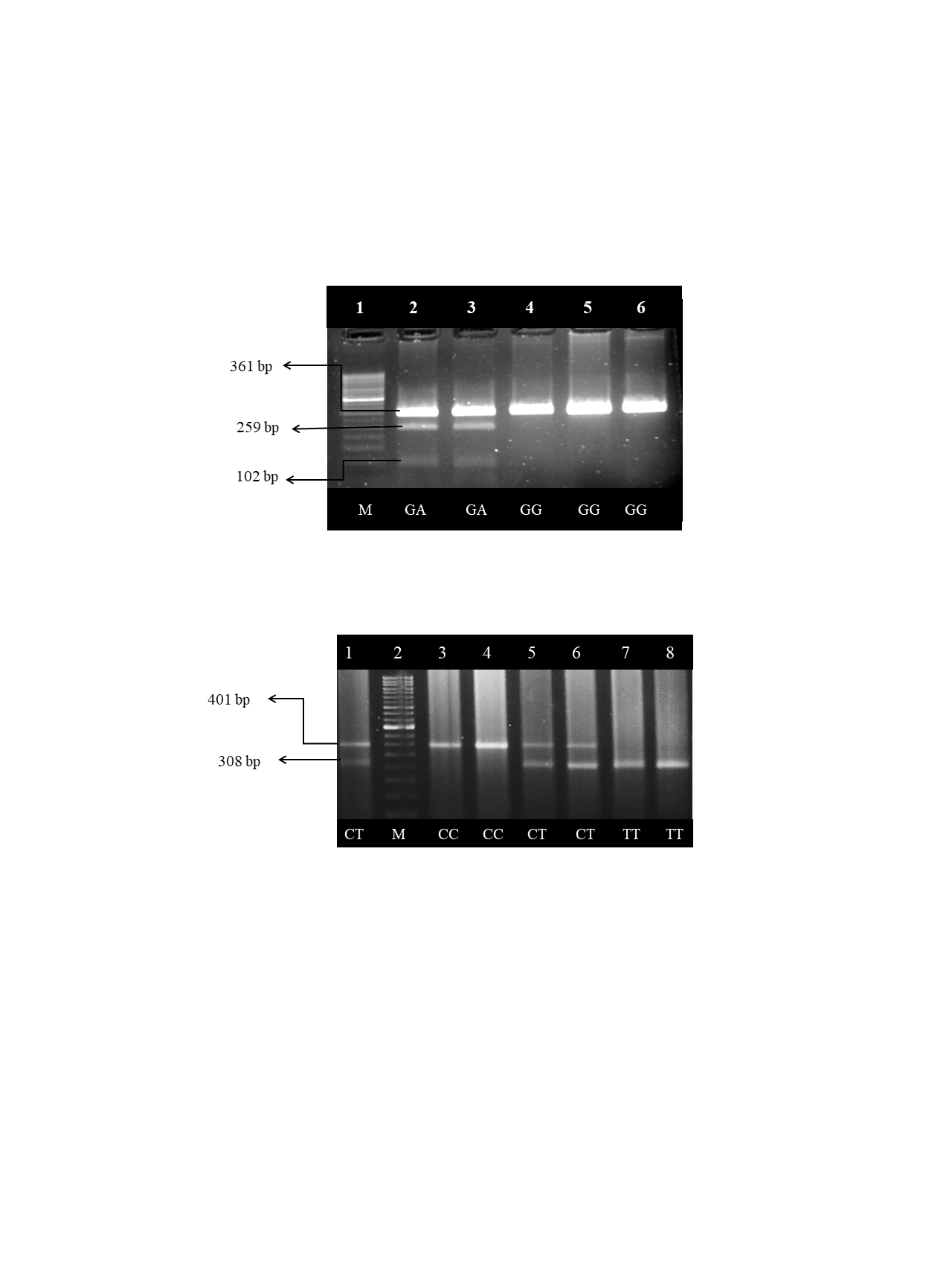
**

**Figure S4: PCR-RFLP analysis of *IL17A* rs8193036 polymorphism on 3.5 % agarose gel electrophoresis.** lanes: 1, 5 & 6 show heterozygous (CT) genotype; lanes: 3 & 4 show homozygous (CC) genotype; lane 7 & 8 show homozygous (TT) genotype; lane 2 shows 50 bp DNA ladder.


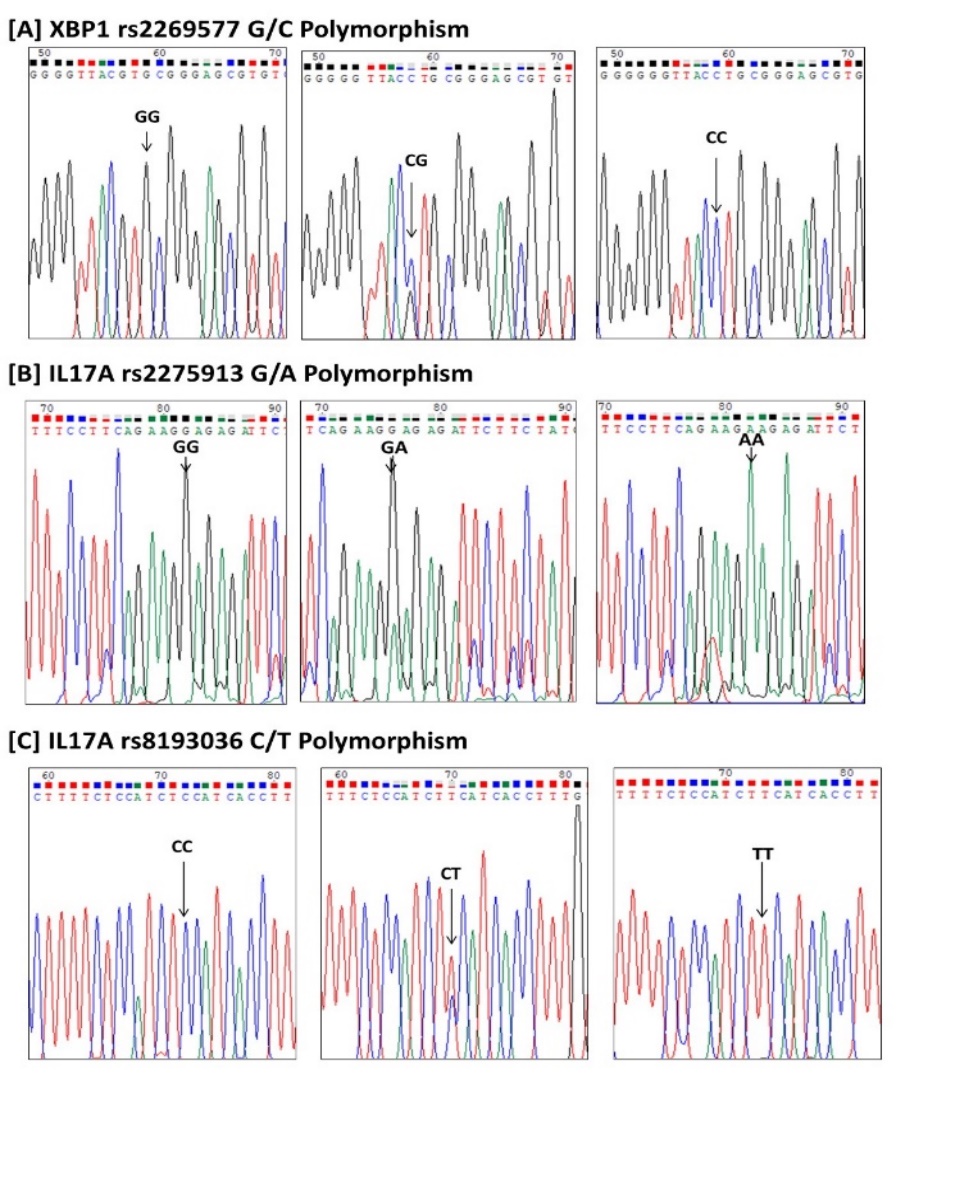


**Figure S5:** Confirmation of genotyping results by Sanger’s sequencing of PCR products. [A] *XBP1* rs2269577 G/C polymorphism, [B] *IL17A* rs2275913 G/A polymorphism and [C] *IL17A* rs8193036 C/T polymorphism.

1. **Supplementary Tables**

**Table S1.** Demographic characteristics of patients with vitiligo and controls.

|  | **Vitiligo Patients** |  | **Controls** |
| --- | --- | --- | --- |
| Average age  (mean age ± SD)  Sex: male  female  Onset age  (mean age ± SD)  Duration of disease  (mean ± SD)  Family history  **Type of disease**  Generalized  Localized  Active  Stable | (n=276) |  | (n=312) |
|  | 32.56 ± 13.25 yrs  149 (53.98%)  127 (46.02%)  22.94. ± 12.78 yrs  6.82 ± 5.43 yrs  84 (30.43%)  204  72  198  78 | 30.64 ± 10.82 yrs  145 (52.54%)  131 (47.46%)  NA  NA  NA  NA  NA  NA  NA | |

**Table S2:** Details of primers and restriction enzymes used for genotyping of single nucleotide polymorphisms and gene expression analysis.

| **Gene/**  **SNP** | **Primer** | **Sequence (5’ to 3’)** | **Annealing**  **Temperature**  **(°C)** | **Amplicon size (bp)** | **RE/ Digested Products (bp)** |
| --- | --- | --- | --- | --- | --- |
| *XBP1*  *rs2269577* | FP | GTTTCAGGACCGTGGCTATG | 60 | 309 | *Tai* I  224 + 85 |
|  | RP | TCGGCTCCACTCGGATC |  |  |  |
| *IL17A*  *rs2275913* | FP | GAGAAAAGAACCGCTAACTCC | 68 | 361 | *Xmn* I  259 + 102 |
|  | RP | TACTCAAGTTCCCATCATATAAG |  |  |  |
| *IL17A*  *rs8193036* | FP | GAGAAAAGAACCGCTAACTCC | 64 | 401 | *Mbo* II  308 + 93 |
|  | RP | TACTCAAGTTCCCATCATATAAG |  |  |  |
| *GAPDH* | FP | CATCACCATCTTCCAGGAGCGAG | 65 | 122 | - |
|  | RP | CCTGCAAATGAGCCCCAGCCT |  |  |  |
| *XBP1* | FP-U  (Unspliced) | TCCGCAGCACTCAGACTAC | 60 | Unspliced:  186  Spliced: 163 | - |
|  | FP-S  (Spliced) | GAGTCCGCAGCAGGTGC |  |  |  |
|  | RP | TGGCAGGCTCTGGGGAAG |  |  |  |
| *IL17A* | FP | ATCACAATCCCACGAAATCCAG | 60 | 188 | - |
|  | RP | CTTTGCCTCCCAGATCACAGAG |  |  |  |
